# Hemolysis-induced hepatic ferroptosis following xenotransfusion of genetically modified pig red blood cells

**DOI:** 10.1038/s41598-025-30021-5

**Published:** 2025-12-16

**Authors:** Ha-Young Shin, Ju Young Lee, Young-Jin Jang, Hyung-Sun Kim, Seon Ryong Park, Goo-Hwa Kang, Joohyun Shim, Nayoung Ko, Jeong Ho Hwang

**Affiliations:** 1 Center for Large Animals Convergence Research, Korea Institute of Toxicology, Jeongeup, Republic of Korea; 2 Center for Bio-Signal Research, Division of Advanced Predictive Research, Korea Institute of Toxicology, 141 Gajeong-ro, Yuseong-gu, Daejeon, 34114 Republic of Korea; 3 Division of Jeonbuk Advanced Bio Research, Korea Institute of Toxicology, Jeongeup, Republic of Korea; 4 Department of Transgenic Animal Research, Optipharm, Inc., Cheongju-si, Chungcheongbuk-do 28158 Republic of Korea; 5 Collage of Veterinary Medicine, Institute of Animal Transplantation, Jeonbuk National University, Iksan-si, Jeonbuk State 54596 Republic of Korea

**Keywords:** Xenotransfusion, Liver injury, Iron overload, Ferroptosis, Diseases, Immunology, Molecular biology

## Abstract

To overcome cross-species immunological barriers, researchers utilize gene editing to remove the expression of three major carbohydrate xenoantigens (*α-Gal*,* Neu5Gc*,* and Sd(a)*) and insert genes, such as human protective genes (*hCD55 and hCD39*). This study aimed to investigate the mechanisms underlying long-term hepatic injury following xenotransfusion of genetically modified pig red blood cells (pRBCs). We xenotransfused pRBCs genetically engineered with GGTA1^−/−^(*α-Gal* ^−)^; CMAH^−/−^(*Neu5Gc*^−^); β4GALNT2^−/−^(*Sd(a)* ^−^*)*; *hCD55; hCD39* into a non-human primate (NHP) model of acute hemorrhage hemolysis and evaluated the long-term immune response to xenogeneic RBCs in the liver, a key organ for RBC metabolism. Immediately after xenotransfusion (D + 1), significant elevations of liver enzymes (AST, ALT) and iron-related factors were observed in the serum levels of the recipients, which were normalized by D + 21. However, long-term analysis revealed excessive accumulation of iron ions in the liver and decreased expression of antioxidant enzymes. The resulting endoplasmic reticulum stress (increased GRP78/BiP expression), lipid peroxide accumulation, and iron-dependent cell death (ferroptosis) persisted in the liver of the recipient. These results suggest that, beyond gene editing, additional hurdles must be overcome to completely block oxidative stress damage caused by impaired RBC lysis. Notably, transcriptomic and immunohistochemical analyses in the NHP model identified the direct role of ferroptosis in xenotransfusion-induced liver injury. Therefore, we propose that a multilayered approach, including blocking the ROS-Iron axis and administering ferroptosis inhibitors, is needed to ensure long-term safety in the clinical development of xenotransfusion.

**Supplementary Information:**

The online version contains supplementary material available at 10.1038/s41598-025-30021-5.

## Introduction

Red blood cell (RBC) transfusion is widely used to restore hemoglobin levels after massive blood loss and improve patient outcomes^1–4^. As worldwide demand for blood and organ transplants continues to rise amid challenges, such as pandemics and aging populations, researchers are exploring xenotransfusion using genetically engineered pig red blood cells (pRBCs), which are similar in size and function to human RBCs^5–12^. However, cross-species immunological barriers and molecular incompatibility pose major obstacles to the clinical application of pRBC transfusion^13,14^. Hemolysis, a key adverse effect, occurs when the recipient’s immune system recognizes xenogeneic RBC antigens as foreign, triggering a strong immune response^15–17^. Genetic engineering approaches have been developed to reduce this response, including eliminating the pig-specific alpha-1,3-galactosyltransferase (GGTA1) and creating triple knockout (TKO) models by deleting genes, such as Cytidine monophospho-N-acetylneuraminic acid hydroxylase (CMAH)^18^, alpha-1,3-Galactosyltransferase 2 (α3GALT2)^19^, or beta-1,4-N-acetylgalactosaminyltransferase 2 (β4GalNT2)^20^, which further reduces immunogenicity^21–24^.

While transfusion of TKO pRBCs in non-human primates (NHPs) has shown improved survival of RBCs compared to wild-type pRBCs, persistence remains limited due to ongoing complement activation and antibody production^20^. Additional modifications, including expression of human complement regulatory proteins, have delayed, but not eliminated, rejection^25^. Despite these challenges, ongoing research into xenogeneic RBC transfusion has provided important insights into immune rejection mechanisms and potential strategies to address them. To overcome cross-species immune barriers and adverse effects and establish the safety and efficacy required for clinical application, further comprehensive studies are essential.

The liver is essential for metabolism and detoxification and is heavily involved in RBC metabolism^26,27^. Normally, iron from senescent RBCs or hemolysis is bound to transferrin and transported to the liver, where it is either stored as ferritin or sent to the bone marrow for new RBC synthesis^28,29^. However, excessive hemolysis releases free hemoglobin, which is degraded into free heme and then into ferrous ions (Fe^2+^) by heme oxygenase-1^30^. Fe^2+^ converts H_2_O_2_ to OH^−^ via the Fenton reaction and causes a spike in reactive oxygen species (ROS) in the liver^31,32^. Antioxidant enzymes (e.g., glutathione peroxidase 4 (GPX4)) are then consumed in large quantities to quench the oxidative stress caused by hemolysis; when their synthesis is impaired, neutralization of ROS fails^33^. This results in secondary damage, which exacerbates oxidative stress in the liver. This oxidative stress promotes lipid peroxidation and ferroptosis, an iron-dependent cell death mediated by ACSL4 activation, contributing to long-term toxicity in metabolic organs, including the liver and kidneys. *GGTA1/CMAH/β4GalNT2* knockout pRBCs persist for approximately 24 h in NHPs^20^, a dramatic increase over the persistence of wild-type pRBCs, which persist for approximately 20 min in the body of NHPs. However, the large degree of RBC hemolysis that occurs after 24 h cannot be ignored in future studies.

To date, the feasibility of xenogeneic RBC transfusion in NHP models has been validated mainly by quantitative recovery of RBCs (Hb, Hct) and analysis of inflammatory markers (ALT, AST), complement activation (C3a, C5a), and immunologic mediators^11,25,34^. However, the physiological functions (antioxidant activity) and metabolic effects (iron distribution and ROS production) of xenogeneic RBC are poorly characterized. Furthermore, the mechanisms of long-term structural damage and cell death caused by hemolysis-induced lysate in RBC metabolizing organs, such as the liver and spleen, after xenogeneic RBC transfusion remain unexplored. Therefore, this study aimed to investigate the mechanisms of long-term liver injury following xenotransfusion of genetically modified pRBCs.

## Materials and methods

### Animal study

#### Experimental animals

A total of seven cynomolgus monkeys (*Macaca fascicularis*, Nafovanny, DongNai Province, Vietnam) were used in this study. The TKO/hGE group had no prior experimental exposure (naïve), and all animals underwent approximately 14 days of acclimation and a pre-treatment period before the experiment.

#### Donor blood preparation

Genetically modified (TKO/hGE) pRBCs, derived from triple-gene knockout pigs lacking GGTA1, CMAH, and β4GALNT2 and additionally expressing human genes (e.g., hCD55, hCD39), were used for xenotransfusion studies. Whole blood was collected from TKO pigs with blood type O (Optipharm Inc., Cheongju, Korea) into blood bags containing acid citrate dextrose (ACD) solution (Changyoung Medical, Chungcheongbuk-do, Korea). The blood was processed through a BioR leukocyte depletion filter (Fresenius Kabi AG, Bad Homburg, Germany) and washed twice with sterile saline. The hematocrit was then adjusted to its original level using SAG-M additive solution (Changyoung Medical). All procedures were conducted under aseptic conditions using a sterile tubing welder (TSCD II, Terumo BCT, Lakewood, CO, USA)^25^.

#### Transfusion procedure

To perform blood collection and transfusion, the TKO/hGE group animals (*n* = 4; 2 males, 2 females) were secured in a primate chair and the blood collection site was disinfected to remove 25% of the total blood volume from the cephalic or femoral vein. After a 1‑hour interval, during which no additional fluid compensation was performed, an equal amount of TKO/hGE pRBCs was slowly transfused into the cephalic vein. Normal control (NC) (*n* = 3; male 1, female 2) were other test animals in the institution that did not receive any treatment and served as controls. The hematologic and serum chemistry markers of NHPs were analyzed before treatment (D + 0 Pre), after blood collection (D + 0 BL), after RBC administration (D + 0 TF), and on days 1, 3, 5, 7, 14, and 21. Observations of general symptoms of the individuals were made daily starting from the day of blood removal/transfusion (D + 0).

#### Clinical pathological analysis

The clinical pathology changes in the TKO/hGE group were analyzed according to the observation period (D + 0 to D + 21). For hematological changes observation, blood was collected in EDTA-2 K tubes, measured, and analyzed by using an ADVIA2120i hematology analyzer (Siemens, USA). For blood biochemical changes observation, blood was collected in SST tubes, centrifuged at 3000 rpm for 10 min, and serum was analyzed for biochemical indices using a TBA 120FR analyzer (Toshiba Co., Japan). Conversely, as the NC group was unable to obtain continuous hematological index data according to the observation period, the hematological and blood biochemistry data of the Saline group, which received an equivalent amount of saline as the blood collection amount during the observation period, were utilized to ascertain whether the alterations in clinical pathological indices were attributable to transfusion. No post-observation autopsies were performed on the saline group, precluding further analysis. Blood parameters in the TKO/hGE and saline groups were evaluated against standard reference ranges using clinical pathology data from normal cynomolgus monkeys (Supplementary Table 2)^35,36^. 

#### Histology

Liver tissues were fixed in 10% neutral buffered formalin, dehydrated, and paraffin embedded. Section (4 μm) were stained with hematoxylin and eosin (H&E) for general histology and neutrophil quantification, and Perl’s Blue for iron deposition (ab150674, Abcam, UK). Stained slides were imaged at 200× magnification using AxioCam and Axio Vision software (Zeiss, Oberkochen, Germany). For quantitative analysis, seven random fields (500 × 500 μm) per animal were assessed for neutrophil and iron-positive cell counts.

#### Immunohistochemistry

Slides were prepared from each animal in each group and immunohistochemical analysis was performed. The slides were deparaffinized, antigens were retrieved, endogenous peroxidase was removed, sections were blocked, primary antibody was incubated, and the secondary antibodies were peroxidase-conjugated. For the peroxidase-conjugated secondary antibody, 3,3’-diaminobenzidine substrate was used, followed by hematoxylin for nuclear counterstaining. Primary antibodies against 4-HNE (1: 100 dilutions, ABIN873270, antibodies), CD68 (1:100 dilution, 76437, cell signaling), GPX4(1:100 dilution, ab125066, Abcam, UK) were used. The image was taken at 200× magnification.

#### Malondialdehyde (MDA) and glutathione (GSH)

Hepatic MDA and GSH levels were measured using the MDA Assay Kit (ab118970, abcam, UK) and GSH and GSSG Assay kit (ab138881, abcam, UK) according to the manufacturer’s instructions.

#### Quantification of total hepatic triacylglycerol levels (TG)

Liver samples were thawed and washed with cold PBS, lysed using a homogenizer. A similar volume of the resulting lysate, corresponding to a similar amount of liver tissue, was used to measure triacylglycerol levels using the Triglyceride Assay Kit (ab65336, abcam, UK) according to the manufacturer’s instructions. To account for the background signal caused by the presence of glycerol in the sample, each sample was calibrated with a background control without the addition of lipase. Measurements were performed at an intensity of 570 nm using a SpectraMax Plus microplate reader (Molecular Devices, Sunnyvale, USA).

#### Immunoblot analysis

Liver tissues were washed with cold PBS, lysed in RIPA buffer with protease/phosphatase inhibitors, and centrifuged at 13,000 rpm for 30 min. Protein concentrations were determined by BCA assay. Equal amounts (10 µg) of protein were denatured, separated on 4–20% SDS-PAGE, and transferred to PVDF membranes. After blocking with 5% BSA, membranes were incubated with primary antibodies and HRP-conjugated secondary antibodies. Bands were detected by chemiluminescence (ChemiDoc, Bio-Rad) and quantified using Image Lab software (Bio-Rad, CA, USA). Primary antibodies used included BiP (3183 S, 1:1000, Cell Signaling), p-IRE1α (PA1-16927, 1:1000, Invitrogen), eIF2α (5324 S, 1:1000, Cell Signaling), p-eIF2α (3398 S, 1:1000, Cell Signaling), CHOP (sc-166682, 1:200, Santa Cruz), α-tubulin (2144 S, 1:1000, Cell Signaling), and ACSL4 (ab155282, 1:1000, Abcam, UK).

#### Quantitative real-time PCR

Total RNA was extracted from liver tissue using the Phenol/Chloroform extraction method and quantified using the Qiexpert system. Complementary DNA was synthesized by reverse transcribing total RNA (1000 ng) using the QuantiTect Reverse Transcription Kit (cat. No. 205311, Qiagen) in a MasterCycler^®^ Nexus GX2 thermal cycler (Eppendorf, Germany). The primer sequences are available in Supplementary Table 1.

#### mRNA sequencing

The construction of cDNA Library was performed using Quantseq 3’ mRNA-Seq Library Prep Kit FWD (Lexogen, Vienna, Austria) according to the manufacturer’s instructions. The process, which included sequencing, mapping, and normalization, was performed in accordance with the manufacturer’s instructions. Differentially expressed genes (DEGs) analysis and data visualization between groups were performed using Excel-based differential expression gene analysis (ExDEGA, Ebiogen Inc., Korea). The determination of DEGs was conducted by identifying genes whose expression levels exhibited as |log2 fold| ≥1.

#### Gene ontology analysis

To compare functional annotations between the Normal and TKO/hGE groups, canonical pathway analysis was performed using IPA (Qiagen, CA, USA). And Gene Set Enrichment Analysis (GSEA) was performed with software version 4.3.3. In order to curate a high confidence signature representative of the live, stringent thresholds of NES FDR < 0.05 were established to signify up- or downregulated genes. The gene sets “h.all.v2025.1.Hs.symbols.gmt”, “c2.cp.kegg_legacy.v2025.symbols.gmt”, and “c2.cp.wikipathways.v2025.1.Hs.symbols.gmt” were obtained from the Molecular Signatures Database (MSigDB). Gene set enrichment analysis (GSEA) for Figs. 2B–E was conducted using these MSigDB gene sets, including KEGG and Wikipathways pathways. KEGG software was not used; KEGG gene sets available in MSigDB were analyzed via the GSEA software.

#### Statistical analyses

Statistical analyses were performed using GraphPad Prism version 8 (GraphPad, San Diego, CA). Data are presented as mean ± standard deviation (SD). Comparisons between groups were performed using the unpaired Student’s t-test. A *p*-value < 0.05 was considered statistically significant.

## Results

### Xenotransfusion-induced hemolysis triggers ER stress and liver injury

To evaluate xenotransfusion effects on the recipient, we performed intravenously infused TKO/hGE RBCs (25% of NHP blood volume;50 mL/kg) into an acute hemorrhage NHP model. Hematological and blood biochemical analyses were monitored over time, and hepatocyte injury, immune cell response, and liver tissue damage were comprehensively evaluated after TKO/hGE pRBCs transfusion.

**Fig. 1:**
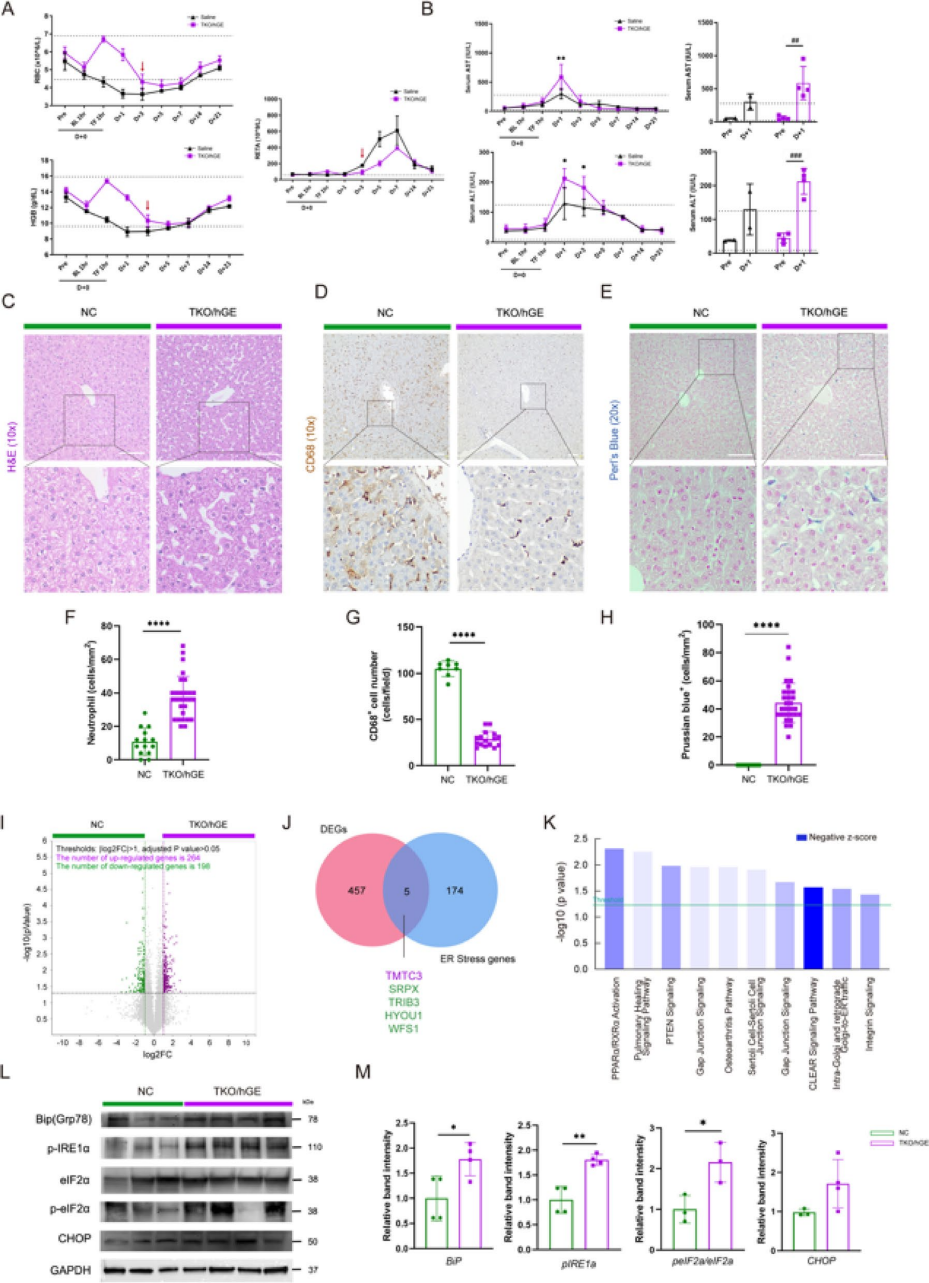
Xenotransfusion-induced hemolysis of TKO/hGE pig RBCs sustains recipient liver injury via ER stress. (**A**) Hematology analysis for RBC-related markers (RBC, HGB, RETA). (**B**) Serum chemistry results for liver injury markers (AST, ALT). Expression of the markers over time (left) and comparison of the levels when they peaked at day D + 1, respectively (right). (**C**–**E**) (**C**) Histopathology (H&E), Red arrows indicate neutrophils. (**D**) Immunohistochemistry (CD68), Yellow arrows indicate CD68^+^ positive cells. (**E**) Perl’s Blue analysis in liver sample. Blue arrows indicate hemosiderin-accumulated cells (scale bar: 100 μm). NC group was not injected (*n* = 3). TKO/hGE group was injected with a single TKO/hGE pRBCs (*n* = 4). (**F**) Volcano plot representation of differentially expressed genes between NC and TKO/hGE liver using RNA-seq analysis. The horizontal and vertical lines indicate the filtering criteria (absolute log_2_FC ≥ 2 and raw adjusted *p*-value < 0.05). The purple and green dots indicate the upregulated (264 genes) or downregulated (198) genes (*n* = 3, *n* = 4 each). (**G**) Venn diagram showing the overlap of DEG in the liver (FC ≥ 2 and raw *p* < 0.05) and ER stress-related genes through GO database. (**H**) Canonical pathway result and negative z-score value in box, (**I**) Immunoblotting for Bip, *p*-IRE1α, eIF2α, p-eIF2α, CHOP in the liver of NC or TKO/hGE NHPs. Density measurement band intensities represent relative values ​​to each control group (*n* = 3 or 4, respectively). For all graphs, the reported values represent mean ± SD. (**p < 0.01, ***p < 0.005)

RBC and Hemoglobin (Hb) levels increased sharply D + 0 Transfusion (TF) compared to D + 0 Blood loss (BL) and decreased significantly on D + 3. This timing coincided with increased numbers of reticulocytes (RETA), suggesting that xenogeneic RBC were largely cleared from the body within 3 days (Fig. 1A, red arrow bar). AST and ALT levels were significantly altered at D + 1 compared to the normal range of healthy animals^36^ (dashed areas of the graph). In the saline group, D + 1 AST and ALT increased approximately 5.7 and 3.4-fold, respectively, compared to the D + 0 Pre. In the TKO/hGE group, mean D + 0 Pre values of AST and ALT increased approximately 10.3-fold and 4.7-fold, exceeding the normal range of healthy animals (dashed area of the graph) (Fig. 1B).

To evaluate the long-term effects of xenotransfusion on the liver, liver tissue was harvested and subjected to histological analysis, despite normalization of hematological values after transfusion. H&E staining revealed significantly increased neutrophil infiltration in the recipient liver tissue after xenotransfusion (Fig. 1C,F) and significantly decreased number of CD68-positive Kupffer cells (Fig. 1D and G). We also observed a significant increase in iron infiltrating cells after xenotransfusion (Fig. 1E,H), possibly owing to the effects of intravascular and extravascular hemolysis. Collectively, these results suggest that, although xenogeneic RBC transfusion transiently achieved functional recovery in the liver, hemolysis due to the immune barrier-induced iron deposition in the recipient organ, despite a single transfusion.

To elucidate the molecular mechanisms of xenotransfusion-induced hemolysis liver injury, mRNA sequencing was performed. Although all clinicopathological values were in the normal range after D + 21, significant gene expression changes were observed in the xenotransfused compared with the NC group. In total, 462 DEGs were selected by statistical criteria (log_2_ Fold change (FC) > 1, *p* < 0.05), of which 264 were upregulated and 198 were downregulated (Fig. 1I). The downregulated gene families were associated with endoplasmic reticulum (ER) stress response by mRNA sequencing. We screened ER stress-related genes through GO databases and identified 5 ER stress genes that overlapped with DEGs identified by strict statistical criteria (log₂FC > 2, *p* < 0.05). Of these, TMTC was upregulated, and SRPX, TRIB3, HYOU1, and WFS1 were downregulated (Fig. 1J). Subsequently, Ingenuity^®^ pathway analysis (IPA^®^, QIAGEN Redwood City) was performed and the top 10 negatively regulated pathways were obtained based on DEG (log2 FC > 2, *p* < 0.05), including downregulation of PPARα/RXRα activation caused by ER stress (Fig. 1K). Significantly increased BiP/GRP78 was observed in the liver tissue of xenotransfused individuals, indicating the molecular triggering mechanism of ER stress following xenogeneic RBC transfusion (Fig. 1L, M). Our results revealed that ER stress, induced by an excessive protein folding load resulting from xenotransfusion, leads to sustained activation of the unfolding protein response (UPR) pathway.

### Xenotransfusion-induced hemolysis inhibits PPARα-mediated fatty acid oxidation

**Fig. 2:**
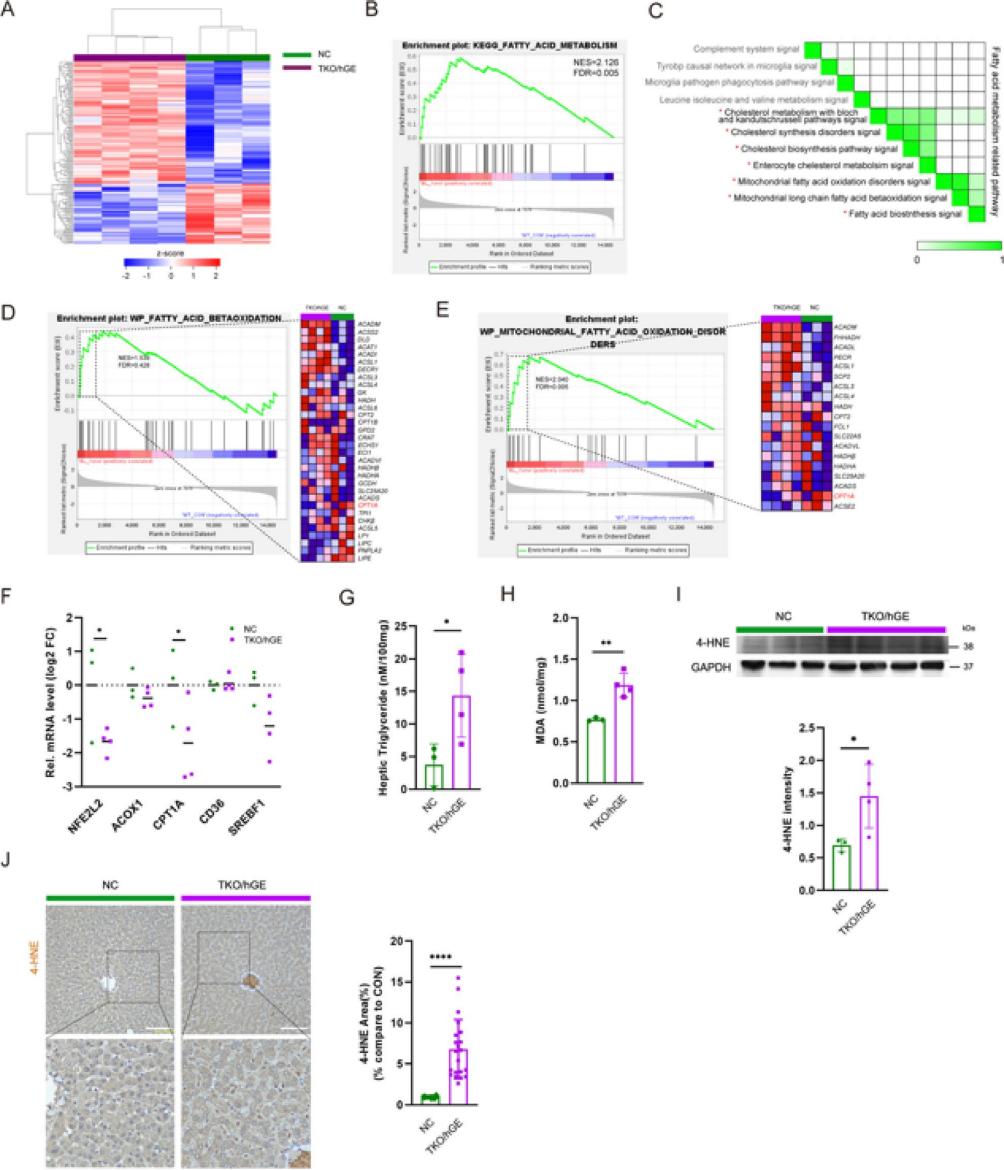
Xenotransfusion-induced hemolysis of TKO/hGE pig RBCs causes fatty acid metabolism dysfunction in recipient liver, leading to fatty acid accumulation. (**A**) Heatmap for differentially expressed genes in liver tissue between NC and TKO/hGE group using RNA-seq data (*n* = 3 or 4). (**B**) GSEA plot of the KEGG categories showing the Fatty acid metabolism (NES (normalized enrichment score) = 2.126, FDR (false discovery rate) = 0.005). (**C**) Leading-Edge analysis of significantly enriched GSEA Wiki pathways. GSEA leading-edge analysis results are shown as a matrix; darker green indicates greater overlap of core genes between gene sets. Fatty acid metabolism-related pathways were indicated with red *asterisks. (**D**,**E**) GSEA plot of the WikiPathway, leading-edge genes showing fatty acid betaoxidation (NES = 1.539, FDR = 0.428) and mitochondrial fatty acid oxidation disorders (NES = 2.040, FDR = 0.005). (**F**) qRT-PCR assays for betaoxidation marker, NFE2L2, ACOX, CPT1A, CD36, SREBF1 in NHPs liver (*n* = 3 or 4). (**G**) Liver Triglyceride (TG) contents (**H**) Malondialdehyde (MDA) levels in NHPs liver. (**I**) Immunoblotting for 4-HNE in the liver of NC or TKO/hGE NHPs. (**J**) Density measurement band intensities represent relative values ​​to each control group (*n* = 3 or 4, respectively). (**K**) Immunohistochemistry for 4-HNE in liver (scale bar: 100 μm). Percent areas of 4-HNE staining were assessed using the Image-J program (*n* = 3 or 4). For all graphs, the reported values represent mean ± SD. Statistical significance was tested via unpaired student’s t-test. (*p < 0.05, **p < 0.01, ***p < 0.005)

Transcriptome heatmap analysis revealed significant changes in liver tissue from TKO/hGE group after xenotransfusion compared with the NC group (Fig. 2A). RNA-seq and GSEA indicated that CHOP-mediated inhibition of PPARα/RXRα activity primarily affected fatty acid metabolism genes (Fig. 2B). Further analysis showed that the top 11 wiki pathways activated lipid metabolism-related pathways, with considerable overlap in lipid metabolism-related pathways and genes (Fig. 2C).

Our previous results identified that PPARα-related pathways, key regulators of fatty acid metabolism, were suppressed in the TKO/hGE group. To elucidate the molecular mechanisms by which the suppression affected the fatty acid metabolism regulatory network, we performed a narrow down-WikiPathway analysis (Fig. 2D, E).

Notably, fatty acid oxidation disorder was increased, whereas CPT1A, which is at the beginning of b-oxidation, was decreased in the recipients. Thus, inactivation of the PPARα/RXRα pathway, associated with mitochondrial fatty acid oxidation disorders, significantly decreased expression of PPARα-dependent fatty acid oxidation-related genes (NFE2L2, CPT1A, CD36, SREBF1) and ACOX genes in the liver by inhibiting fatty acid beta-oxidation (FAox) (Fig. 2F). This led to the accumulation of undegraded fatty acids and significantly increased liver TG levels (Fig. 2G). Inhibition of FAox caused intracellular free fatty acids to accumulate, promoting oxidative stress and fatty acid peroxidation (Fig. 2H), ultimately resulting in increased production of 4-hydroxynonenal (4-HNE) (Fig. 2I, J). Thus, inhibition of PPARα/RXRα axis activation decreased FAox-related gene expression, which reduced fatty acid oxidation capacity in mitochondria and peroxisomes, leading to lipid metabolic abnormalities accompanied by a significant increase in intracellular TGs and lipid accumulation. These results suggest that metabolic changes induced by xenotransfusion may serve as risk factors for various metabolic diseases.

### Xenotransfusion-induced hemolysis leads to ROS imbalance and glutathione depletion, causing ferroptosis in the liver

**Fig. 3:**
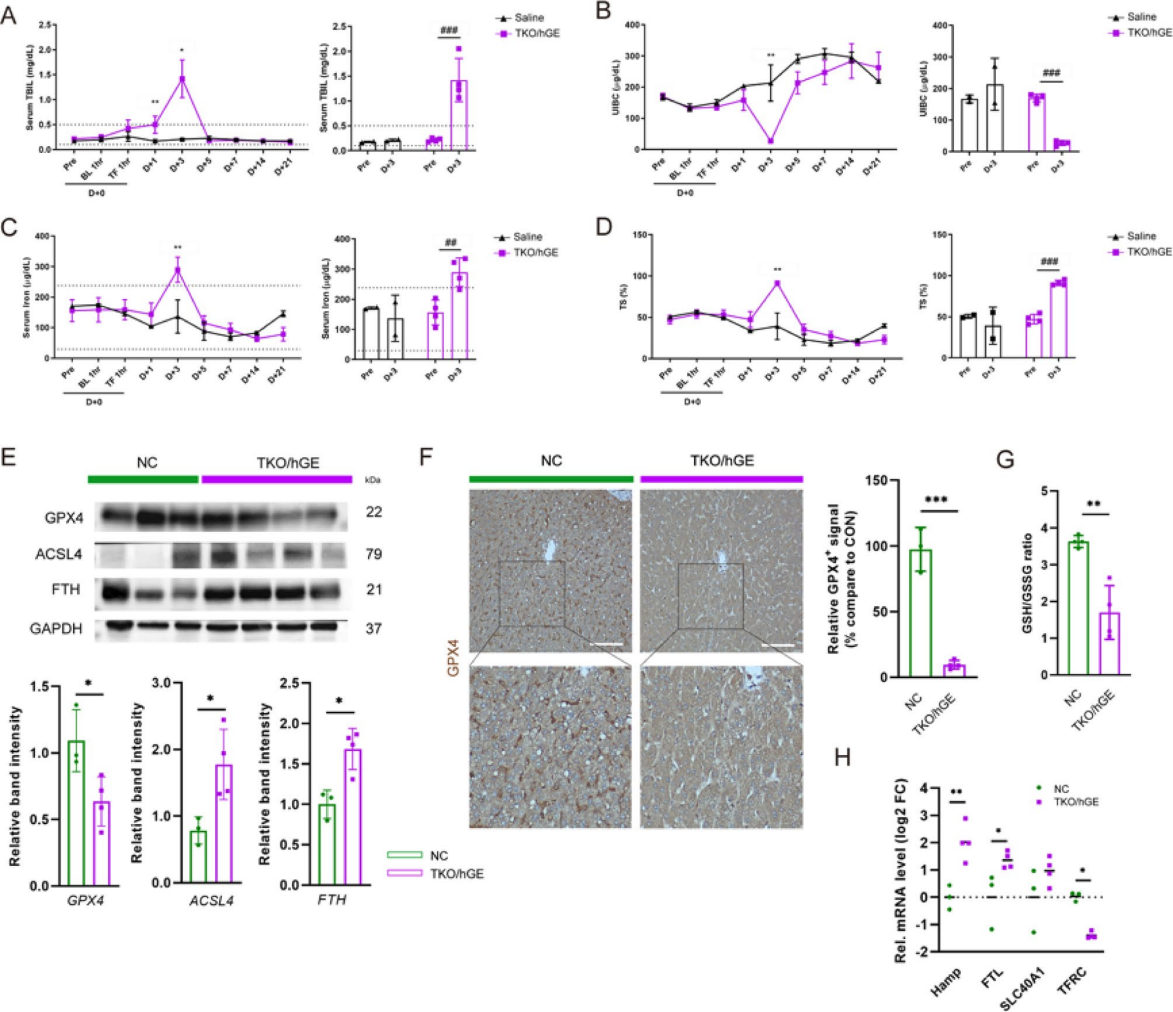
Xenotransfusion-induced hemolysis of TKO/hGE pig RBCs causes massive iron release, glutathione depletion, leading to ferroptosis in recipient liver. (**A**–**D**) Serum chemistry results relevant to iron metabolism disorders and hemolysis-associated markers. Expression of the markers over time (left) and comparison of the levels when they peaked at day D + 3, respectively (right). (**A**) Serum total bilirubin (TBIL), (**B**) Unsaturated Iron Binding capacity (UIBC), (**C**) Serum Iron, (**D**) Transferrin saturation (TS). The dotted area indicates the normal range. *: a significant difference compared to the saline group, #: a significant difference compared to the pre-state within each group. (**E**) Immunoblotting for GPX4, ACSL4, and FTH in the liver of NC or TKO/hGE groups. Density measurement band intensities represent relative values ​​to each control group (*n* = 3 or 4, respectively). (**F**) Immunohistochemistry for GPX4 in liver (scale bar: 100 μm). Percent areas of GPX4 staining were assessed using the Image-J program (*n* = 3 or 4). (**G**) Oxidized/reduced glutathione ratio (GSH/GSSG) in liver. (**H**) qRT-PCR assays for iron metabolism marker, Hepcidin (Hamp), Ferritin L(FTL), SLC40A1, TFRC (Transferrin Receptor Protein 1, CD71) in NHPs liver (*n* = 3 or 4). For all graphs, the reported values represent mean ± SD. Statistical significance was tested via unpaired student’s t-test. (*p < 0.05, **p < 0.01)

Xenogeneic RBC degraded in the recipient 1‒3 days after a single transfusion^20^. To determine the effect of excess lysate released from degraded RBCs on hepatic iron metabolism, we identified blood biochemical markers related to iron metabolism after transfusion. Levels of serum Total bilirubin (TBIL), serum Iron (Fe), total iron binding capacity (TIBC), transferrin saturation (T/S), and unsaturated iron binding capacity (UIBC) in the xenogeneic recipients were significantly altered at D + 3 post-xenotransfusion compared with the normal range (dashed area of the graph)^36^ and before xenotransfusion. Specifically, in the TKO/hGE group, D + 3 serum TBIL increased approximately 6.6-fold compared to the D + 0 pre, which exceeds the normal range (dashed area of the graph, Supplement Table 2). Concurrently, serum iron concentration increased by approximately 1.9-fold, T/S concentration increased by approximately 1.9-fold, and UIBC decreased by approximately 6.3-fold compared D + 0 pre. Conversely, in the saline group, serum iron levels and T/S ratio were at 0.8-fold, UIBC level exhibited a 1.3-fold increase. Levels of TIBC remained consistent in both groups, indicating a secondary iron overload resulting from RBC lysis that occurred in the TKO/hGE group following transfusion. (Fig. 3A–D) We first measured the tissue expression of GPX4, a key enzyme in GSH metabolism^37^, observing a significant decrease in GPX4 in the liver following xenotransfusion (Fig. 3E–G). Consistent with these results, we observed that the GSH/GSSG ratio between xenogeneic recipients was almost half of the ratio detected in NC livers (Fig. 3F). This implies a potential redox imbalance and higher vulnerability to oxidative stress in response to xenotransfusion.

To understand the mechanisms of iron overload and oxidative stress-induced ferroptosis following hemolysis, we examined the expression of ACSL4, a key enzyme in ferroptosis. We observed a significant increase in ACSL4, FTH protein expression in the liver of xenogeneic RBC recipients (Fig. 3E) and an increase in ferroptosis and related enzymes involved in iron metabolism (Hamp, FTL, SLC40A1, and TFRC), confirming that iron metabolism dysregulation occurs in the liver after xenotransfusion, resulting in ferroptosis induction (Fig. 3H).

## Discussion

Recent xenotransfusion research focuses on improving the survival of pRBCs in recipients;^10,20^ however, the mechanisms and risks of hemolysis-related organ damage remain unclear. As a recent study in 2024 identified the “lack of assessment of hemolysis-related organ damage” as a major limitation^38^, long-term safety and risk assessments at the preclinical stage remain key challenges. In this study, to primarily protect against acute immune rejection, genetically modified pRBCs (*GGTA1*^−/−^, *CMAH*^−/−^, *β4GALT2*^−/^;*hCD55;hCD39*) were administered via a single transfusion to NHP that had experienced massive hemorrhage.

Although recipient clinical pathology was recovered within three weeks, the liver showed lasting tissue damage, ongoing UPR, inhibited fatty acid metabolism, and induced ferroptosis. These results demonstrate that hemolysis after xenotransfusion can cause long-term liver injury, not only oxidative stress and iron overload, but also ferroptosis via the ER stress/fatty acid metabolism pathway. Hemolysis byproducts promote harmful lipid peroxidation and weaken the body’s antioxidant defenses in the liver. This study highlights the need for improved safety monitoring, new biomarkers, and targeted therapies (iron chelators or antioxidants) to reduce long-term risks in future xenotransfusion approaches.

Our results clearly indicate biochemical evidence consistent with hemolysis after xenogeneic pRBC transfusion in NHPs. This condition stems from strong immune reactions, particularly involving the complement system, which rapidly destroys the transfused pRBCs^39–41^. This leads to the release of large amounts of hemoglobin, heme, and iron. Our findings definitively link this to acute liver injury, oxidative stress, and inflammation^42–44^. This is evident from elevated liver enzymes and evidence of cell damage in our study. Heme-derived iron (Fe²⁺) generates ROS via the Fenton reaction^45^, causing mitochondrial dysfunction, DNA damage, and protein modification^32,46^. Notably, ROS-induced protein damage also causes ER stress and activates the UPR^47^, as shown by the persistent elevation of BiP/GRP78 and UPR pathway activation (PERK-eIF2α, IRE1α-XBP1, ATF6). Although liver function and blood values recover within three weeks, these molecular changes indicate long-term disruption of protein homeostasis and sustained ER stress, highlighting the need for continuous monitoring of molecular and histological markers to ensure the long-term biocompatibility and safety of xenotransfusion.

The ER is crucial for lipid synthesis and metabolism, as it contains key enzymes and regulatory proteins (e.g., ACLY, FASN, SREBP-1c)^48,49^. When the liver experiences ER stress, it activates the PERK/eIF2α signaling pathway^48–50^. This activation increases stress-related proteins (ATF4, CHOP) and suppresses the PPARα/RXRα pathway, which normally regulates genes involved in FAox and antioxidant defense^51,52^. Several studies have demonstrated the mechanism by which inhibition of the PPARα/RXRα pathway leads to TG accumulation^52,53^. Inhibition of the PPARα/RXRα pathway increases FATP1 expression and triglyceride accumulation, while its activation, such as with PPARα agonists, reduces these effects and protects against iron overload, ferroptosis, and lipid peroxidation. The PPARα/RXRα pathway protects the liver by regulating fatty acid oxidation and antioxidant defenses in macrophages^52^. PPARα knockout mice^51^, and transfusion-induced iron deposition models^54^. RNA-seq analysis of liver tissue from xenotransfused non-human primates revealed significant changes in genes involved in fatty acid metabolism, specifically showing suppression of the PPARα/RXRα pathway. This suppression led to decreased expression of key fatty acid oxidation genes (CPT1A, CD36, SREBF1, ACOX1), resulting in triglyceride accumulation and impaired fatty acid breakdown. Consequently, excess fatty acids were oxidized by ROS, increasing markers of oxidative stress (4-HNE, MDA) and contributing to liver injury. These results suggest that xenotransfusion disrupts hepatic lipid metabolism and increases oxidative damage through inhibition of the PPARα/RXRα pathway; activating this pathway could be a promising strategy to prevent or treat secondary liver injury. However, further studies are needed to clarify these mechanisms and their therapeutic effects.

In patients with blood disorders requiring constant transfusions (e.g., aplastic anemia and myelodysplastic syndromes), repeated transfusions lead to excessive accumulation of iron in the body^55^. Iron overload induces intracellular oxidative stress by promoting ROS production^56^ and lipid peroxidation^57^. Decreased ratio of the concentration of reduced and oxidized GSH and GSH/GSSG ratio is an indicator of cytotoxicity that can occur under oxidative stress conditions. Excess iron catalyzes the formation of ROS via chemical processes like the Fenton reaction, leading to increased oxidative stress within hepatocytes^58^. This oxidative stress depletes reduced GSH and shifts the GSH/GSSG redox balance toward the oxidized state, impairing the cell’s antioxidant defense^37,59^. The high redox activity of iron further exacerbates cellular injury, ultimately promoting hepatocellular damage and cell death^32,60^. Consistent with this concept, a growing body of evidence supports that excess iron contributes to liver damage. Iron accumulation in the body due to antioxidant imbalance can affect major organs, such as the kidneys and liver, with liver cell damage being particularly prominent owing to persistent iron accumulation in the liver. Iron toxicity increases tissue damage in an animal model of hemolysis-induced oxidative stress^61,62^. Accumulation of iron in the liver of animal models causes downregulation of hepatic PPARα-Sirt3-Wnt signaling, which accelerates liver fibrosis^54^. In patients with congenital hemolytic anemia, iron excess can occur due to increased iron absorption and persistent hemolysis, even without transfusion, which triggers iron-dependent ferroptosis, promoting liver damage and fibrosis^63,64^.

Ferroptosis, an iron-dependent form of regulated cell death, is characterized by a marked decline in glutathione levels and a surge in lipid peroxidation, particularly in conditions of chronic hemolysis or transfusion^65^. Initially, iron from lysed red blood cells is processed by Kupffer cells, but persistent overload exceeds their capacity, resulting in iron accumulation in hepatocytes^66,67^. This triggers further oxidative stress, lipid peroxidation, and ferroptosis, causing secondary liver damage^68,69^. While these mechanisms are increasingly recognized, the precise pathways of ferroptosis in the liver after xenotransfusion remain to be fully elucidated. Here, we observed that hemolysis after xenotransfusion leads to increased iron production in the liver. We aimed to elucidate the mechanisms of secondary liver injury after hemolysis and contribute to the development of targeted therapeutic strategies by characterizing the molecular nature of ferroptosis under these conditions. When genetically engineered xenogeneic RBC preparations were administered to NHPs, hematological and biochemical analyses revealed elevated levels of liver-related metabolic enzymes. Additionally, hemosiderin accumulation was detected in hepatocytes, suggesting that hemolysis after xenotransfusion is directly linked to iron accumulation in the liver. These results suggesting the hypothesis that hemolysis induced by xenotransfusion leads to long-term iron accumulation in the liver and sustained ferroptosis. Furthermore, our findings suggest that lipid metabolism and ER stress, key markers of ferroptosis, are potential therapeutic targets for mitigating liver injury in this context. The correlative between iron excess, lipid peroxidation, and ER stress underscores the complexity of ferroptosis-related liver injury and highlights the need for a multifaceted therapeutic approach. Interventions aimed at restoring glutathione homeostasis, inhibiting lipid peroxidation, or modulating ER stress pathways could alleviate ferroptosis and reduce liver injury after hemolysis, for example. This integrative perspective advances our understanding of the molecular mechanisms of post-xenotransfusion liver injury and paves the way for novel, mechanism-based therapies.

Another important consideration for clinical translation involves the distinction between intravascular hemolysis, as modeled in our study, and the predominantly extravascular hemolysis anticipated in xenotransfusion recipients^70,71^. The extravascular hemolysis, driven by hepatic and splenic macrophages, is expected to remove transfused pRBCs more gradually, potentially resulting in a chronic pattern of iron accumulation and long-term hepatic stress mechanisms. Experimental studies in murine model support this pathway, showing that hepatic and splenic macrophages mediate gradual clearance of RBCs and iron, with measurable increases in hepatic iron content and markers of oxidative stress overtime^27,72^.​

The relative kinetics and severity of hepatic ferroptosis may therefore differ substantially between these two methods of hemolysis, with acute hemolysis favoring pronounced metabolic and histological injury^73^, and slower macrophage-driven process being associated with persistent but less severe consequences^74,75^. Thus, both innate (IgM) and adaptive (IgG) immune responses may play roles in this process, even in clinically crossmatch-negative settings^76^. To further illustrate these mechanistic differences and their translational implications, a comparison of intravascular hemolysis versus extravascular hemolysis hepatic responses is provided.(Supplementary Table 3) Such comparative analysis underscores the need for dynamic in vivo studies and functional assays to optimize risk management and guide the development of targeted therapies for xenotransfusion safety.

This study is subject to several limitations. First, necropsy was not performed on animals that received saline after blood loss, so this group was assessed only by clinical pathology. For tissue and molecular analyses, healthy, untreated animals were used as NCs; however, this may not fully reflect the effects of blood loss and saline infusion and should be considered when interpreting the results. Second, despite anatomical and physiological similarities between NHPs and humans, this study used human databases for GSEA due to limited NHP-specific resources. While this leverages genetic conservation, it may not fully capture species-specific differences, potentially affecting the accuracy of enrichment results. To address this, we also performed IPA analysis using non-human primate datasets to improve the reliability and biological relevance of our pathway interpretations. Third, while our transcriptomic data focused on long-term outcomes, the inability to capture acute-phase molecular events limits our understanding of the initial triggers of hemolysis-driven ferroptosis. However, the observed long-term pathological outcomes (e.g., iron overload, lipid peroxidation) and their association with clinical parameters remain robust, supporting the proposed therapeutic strategies.

## Conclusion

Thus, this study indicates that effective management of hemolysis after xenotransfusion is crucial for regulating hepatic iron metabolism and improving patient prognosis, as illustrated by three main findings. First, while short-term hematologic and biochemical improvements occur after xenotransfusion, long-term tissue responses reveal persistent risks. Second, xenotransfusion triggers immune-mediated hemolysis, highlighting the need for robust primary interventions. Complement blockade strategies such as eculizumab (anti-C5) and Cp40 (C3 inhibitor) can effectively attenuate acute, complement-mediated hemolysis, as supported by recent preclinical and translational studies. Additionally, beyond complement regulation with hCD55 and hCD39, long-term strategies targeting extravascular hemolysis—such as macrophage checkpoint engineering with hCD47—are warranted to address chronic hemolytic complications. Finally, this mechanistic sequence underscores the necessity for comprehensive strategies in the clinical application of genetically modified RBCs. Specifically, gene editing is employed as a first-line intervention to overcome interspecies immunological barriers and suppress immune-mediated hemolysis. Nonetheless, secondary organ injury driven by hemolysis-induced iron overload, ferroptosis, and ROS-mediated stress should be addressed through additional targeted approaches. Without such dual-layered management, excessive iron accumulation following hemolysis may result in progressive hepatocellular damage, liver failure, and poor clinical outcomes. These findings provide a foundation for targeted therapies and highlight the need for further research into the role of TKO/hGE pRBCs in xenotransfusion-related pathogenesis and the development of multifaceted solutions.

## Supplementary Information

Below is the link to the electronic supplementary material.

Supplementary Material 1

Supplementary Material 2

Supplementary Material 3

## Data Availability

Original data are available on request from the corresponding author, Jeong Ho Hwang (jeongho.hwang@kitox.re.kr). The raw mRNA sequencing data generated and analyzed in this study have been uploaded in the NCBI Gene Expression Omnibus (GEO) under accession number GSE306520 at https://www.ncbi.nlm.nih.gov/geo/query/acc.cgi?acc=GSE306520.
