# Supplementary figures and images for: Hemolysis-induced hepatic ferroptosis following xenotransfusion of genetically modified pig red blood cells
Source: Sci Rep. 2025 Dec 16;15:44014. doi: 10.1038/s41598-025-30021-5 (PMC12711946; doi:10.1038/s41598-025-30021-5)

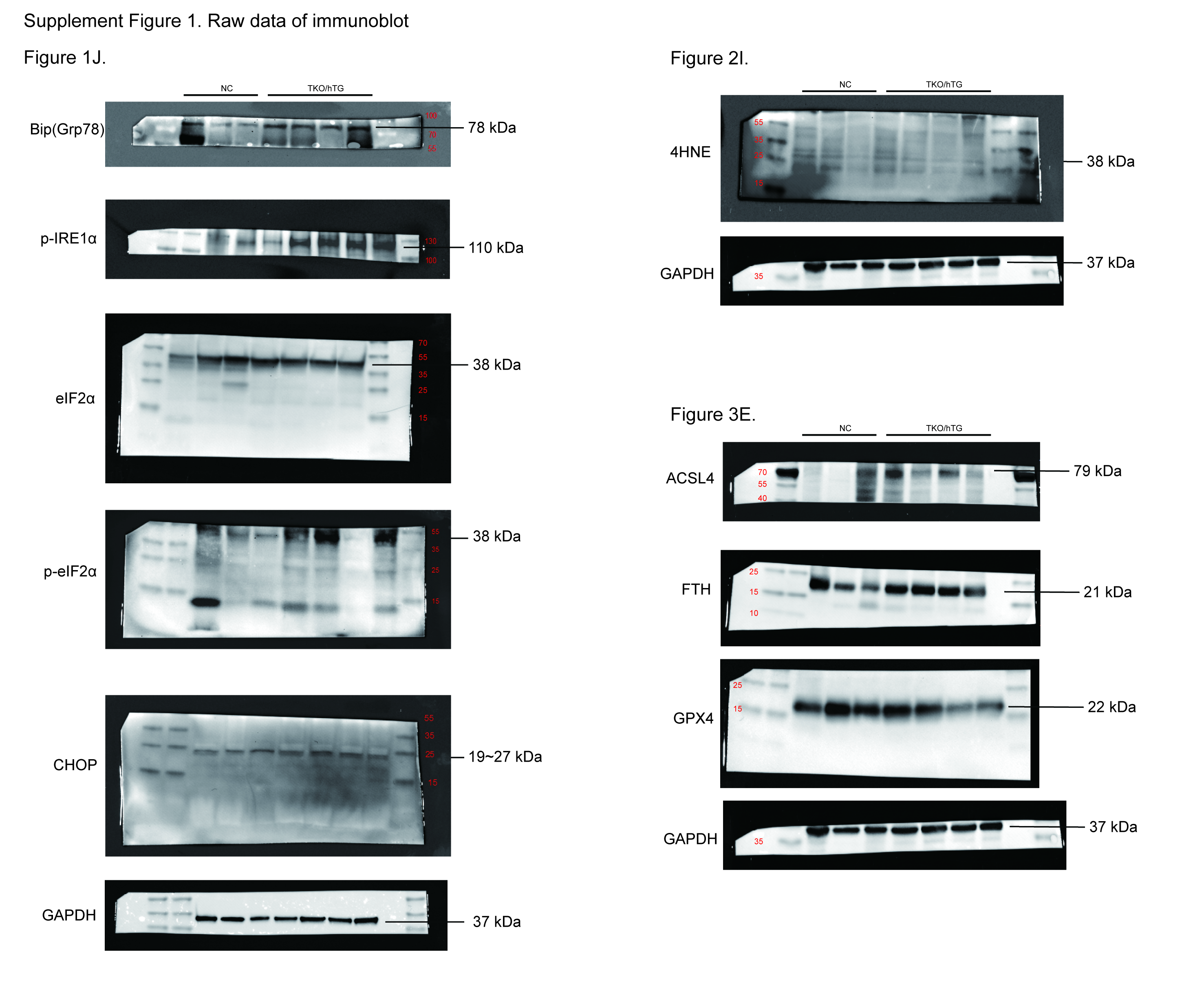

Supplement: Supplementary file 1 — Supplementary Material 1 [file 41598_2025_30021_MOESM1_ESM.tif]
