# Supplementary material for: Hemolysis-induced hepatic ferroptosis following xenotransfusion of genetically modified pig red blood cells
Source: Sci Rep. 2025 Dec 16;15:44014. doi: 10.1038/s41598-025-30021-5 (PMC12711946; doi:10.1038/s41598-025-30021-5)
Supplement: Supplementary file 2 — Supplementary Material 2 [file 41598_2025_30021_MOESM2_ESM.docx]

**Supplement Table 1.** The sequence of primers

| **Gene symbol** | **Primer sequences (from 5’ to 3’)** | **Length** | **Gene Bank ID** |
| --- | --- | --- | --- |
| *NFE2L2* | F: AAACCAGTGGATCTGCCAAC | 135 | XM_005573572.4 |
|  | R: ACGTAGCCGAAGAAACCTCA |  |  |
| *ACOX1* | F: GTGAAGCCTGATGGCACATA | 94 | XM_074020572.1 |
|  | R: CAGCTTCTCCCACAAGGAAG |  |  |
| *CPT1A* | F: CATCATCACTGGCGTGTACC | 102 | XM_065527846.1 |
|  | R: ATTCCTAACGAGGGGTCGAT |  |  |
| *CD36* | F: TCCAGTTCAAAACCCAGACA | 112 | XM_005550372.5 |
|  | R: CACAGGTTTCCCTTCTTTGC |  |  |
| *SREBF1* | F: GACAGACGGAACCACTGTGA | 139 | XM_065532293.1 |
|  | R: CTTGTCCGCATCTACAACCA |  |  |
| *Hamp* | F: CGATCAAAGTGTGGGATGTG | 87 | XM_005588836.4 |
|  | R: GGGGCAGCAGGAATAAATAA |  |  |
| *FTL* | F: AAGGCATGAGCCACTTCTTC | 122 | XM_074024136.1 |
|  | R: AGCTGGCTTCTTGATGTCCT |  |  |
| *SLC40A1* | F: GCTACTGCAATCACGATCCA | 90 | XM_005573701.4 |
|  | R: TCGTATGGTGGCATTCATGT |  |  |
| *TFRC* | F: GACATGCTCATCTGGGAACA | 125 | XM_045387277.2 |
|  | R: AGCTCTGGAGATCGTTTGGA |  |  |
| *GAPDH* | F: TGTTGCCATCAATGACCCCT | 120 | XM_074006169.1 |
|  | R: TTGATGACGAGCTTCCCGTT |  |  |

**Supplement Table 2.** Normal range of clinical pathology parameters in cynomolgus monkeys

| **Parameter** | **Unit** | **Reference normal range** | **Reference** |
| --- | --- | --- | --- |
| RBC | 10^6^/µL | 5.0 – 6.6 | [35] |
| HGB | g/dL | 11.7 – 15.7 | [35] |
| RETA | 10^9^/L | 32.6 – 170.7 | [35] |
| Serum AST | IU/L | 26.6 – 189.0 | [35] |
| Serum ALT | IU/L | 11.0 – 230.3 | [35] |
| Serum TBIL | mg/dL | 0.1 – 0.5 | [35] |
| UIBC | µg/dL | - | - |
| Serum Iron | µg/dL | 29.6 ~ 238.0 | [36] |
| TS | % | - | - |
