# Supplementary material for: Hemolysis-induced hepatic ferroptosis following xenotransfusion of genetically modified pig red blood cells
Source: Sci Rep. 2025 Dec 16;15:44014. doi: 10.1038/s41598-025-30021-5 (PMC12711946; doi:10.1038/s41598-025-30021-5)
Supplement: Supplementary file 3 — Supplementary Material 3 [file 41598_2025_30021_MOESM3_ESM.docx]

**Supplement Table 3.** Mechanistic potential differences between intravascular and extravascular hemolysis in xenotransfusion.

| **Characteristic** | **Intravascular hemolysis** | **Extravascular hemolysis** |
| --- | --- | --- |
| **Primary clearance pathway** | Complement | Macrophage phagocytosis |
| **Speed of RBC clearance** | Rapid,  within hours to days | Slow,  persistent over to weeks |
| **Major immune mediators** | Pre-existing antibodies, complement | Fc receptor-mediated phagocytosis (IgG/IgM) |
| **Hepatic iron overload pattern** | Sudden, massive | Chronic, incremental |
| **Oxidative stress** | Acute, high-intensity | Chronic, low-intensity |
| **Hepatic ferroptosis** | Pronounced, acute | Slight, long-term |
| **Histological changes** | Severe hepatocellular injury | Mild, protracted inflammation/fibrosis |
| **Clinical implication** | Immediate risk: acute failure | Long-term risk: subclinical liver dysfunction |
| **Translational** | Emergency interventions,  iron chelation | Surveillance,  chronic risk mitigation |
